# Supplementary material for: Ultrafast unidirectional spin Hall magnetoresistance driven by terahertz light field
Source: Nat Commun. 2025 Mar 6;16:2249. doi: 10.1038/s41467-025-57432-2 (PMC11885675; doi:10.1038/s41467-025-57432-2)
Supplement: Supplementary file 1 — Supplementary Information [file 41467_2025_57432_MOESM1_ESM.pdf]

Supplementary Information: Ultrafast unidirectional spin Hall magnetoresistance driven by terahertz light field

Ruslan Salikhov[1*], Igor Ilyakov[1], Anneke Reinold[2], Jan-Christoph Deinert[1], Thales V.A.G de Oliveira[1], Alexey Ponomaryov[1], Gulloo Lal Prajapati[1], Patrick Pilch[2], Ahmed Ghalgaoui[2], Max Koch[2], Jürgen Fassbender[1,3], Jürgen Lindner[1], Zhe Wang[2], Sergey Kovalev[2*]

[1]Helmholtz-Zentrum Dresden-Rossendorf, Dresden, Germany.
[2]Department of Physics, TU Dortmund University, Dortmund, Germany.
[3]Institute of Solid State and Materials Physics, TU Dresden University, Dresden, Germany.

*Corresponding author(s). E-mail(s): r.salikhov@hzdr.de; sergey.kovalev@tu-dortmund.de;

1 Experimental setup

Figure 1 illustrates the experimental setup used for the THz SHG characterization. The THz pump pulses, operating at frequency Ω, were produced by the superradiant THz source, TELBE, located at the Helmholtz-Zentrum Dresden-Rossendorf. An additional set of THz bandpass filters (BP_{Ω}) with a center frequency of Ω was added to the THz beam path to clean the spectrum and eliminate extraneous radiation beyond the fundamental band. The maximum peak field strength of the THz radiation focused on the sample was set at approximately 100 kV/cm. Three wire grid polarizers (WG_1, WG_2, WG_3) were used to manipulate both the amplitude and polarization direction of the THz pump pulses.

These polarizers were mounted on computer-controlled motorized rotational stages, allowing for precise adjustments. The superradiant THz source generates a THz field with vertical polarization, as defined in the main text, Fig. 1. The WG_1 polarizer was used to clean the THz polarization. Using WG_3, we set the polarization angle

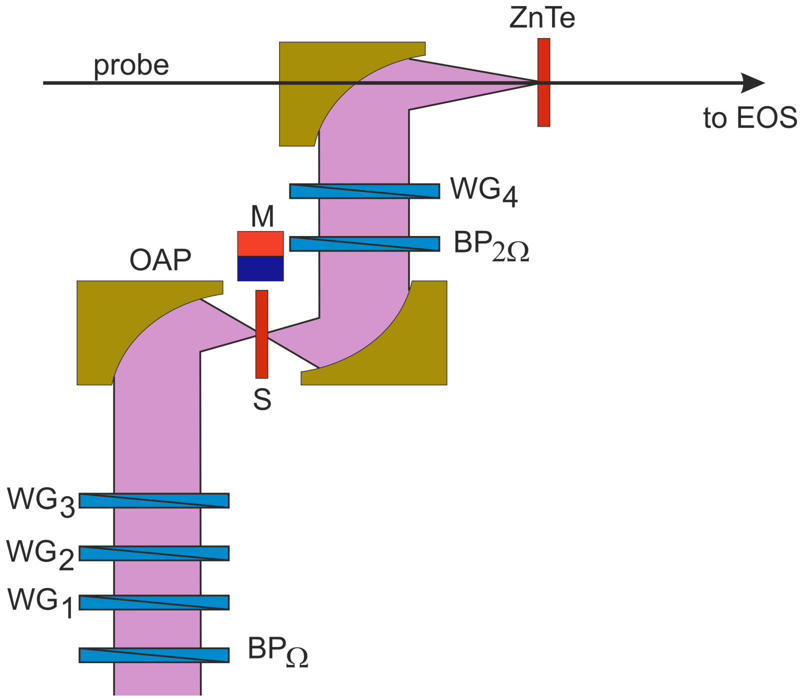

Fig. 1 Sketch of the experimental setup of THz SHG.

of the THz pulse incident on the sample surface. Meanwhile, WG_2 was utilized to regulate the amplitude of the THz pump, ensuring it remained constant across all polarization angles used in the angular dependencies. Calibration of both the angles and the THz pump field strength was conducted prior to the experimental runs. To control the ellipticity of the THz beam, a THz quarter-wave plate was positioned after the WG_3. The THz beam was focused onto the sample using the off-axis paraboloidal mirrors (OAP). The sample's magnetization was aligned with the external magnetic field generated by a NdFeB permanent magnet (M) 50x30x12 mm in size. The resulting magnetic field at the sample position was measured to be approximately 100 mT.

After the sample, a pair of bandpass filters was used to allow THz transmission at a frequency of 2Ω. The WG_4 was used to control the polarization of the transmitted beam. Subsequently, the beam was refocused onto the ZnTe crystal, and its temporal profile was examined using standard electro-optical sampling (EOS) with laser pulses of 35 fs duration at a central wavelength of 800 nm. The samples investigated were grown on a 5x5 mm or 10x10 mm fused silica substrate with a thickness of 1 mm. In both cases, the THz pump pulse focused on the sample had a diameter of less than 1 mm, and the sample cross-section did not affect the fundamental THz and SHG beams propagation. Due to the limited operating time of the TELBE facility, the temperature dependence of $E^{(2\Omega)}_{USMR}$ (Figure 4b) was performed using a laser-based THz source. Tilted pulse front THz generation was used instead of the TELBE source. Due to the low repetition rate and low spectral density of the laser-based source, as

well as additional losses of the THz beam at the cryostat windows, the signal-to-noise ratio of the THz SHG is only 10 at room temperature, and we could not resolve the signal at temperatures below 50 K.

To improve the statistics and signal-to-noise ratio of the THz SHG signals, while accounting for potential fundamental power drift during time delay scans and minimizing the impact of parasitic fundamental radiation, we conducted four delay scans per sample magnetization with opposite directions. Exploiting the asymmetric behavior of the SHG signal under the magnetization inversion, we took the difference between the signals for opposing magnetization directions. This approach enables us to minimize parasitic contributions to the detected THz SHG signal that are not related to spintronic effects.

The EOS and WG_4 were adjusted to detect the horizontal polarization component of the THz SHG signal for the THz pump polarization and ellipticity dependencies. In the vertical polarization detection scheme, the SHG signal experienced an additional parasitic contribution from the vertically polarized THz radiation emitted by the source. Along with the time constraints of the user facility, this limited our ability to explore the SHG polarization angular dependence.

2 THz SHG amplitude dependence

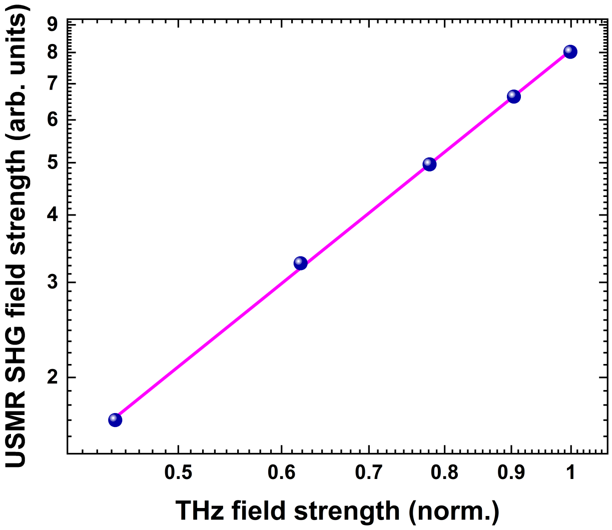

Fig. 2 Dependence of the THz SHG field strength on the field strength of the fundamental radiation in the Ta(2nm)/Py(3nm)/Pt(2nm) sample. The data points were obtained in the horizontal magnetisation geometry at a THz pump polarisation angle of $\phi = 45°$, ensuring that only the USMR-SHG signal was detected. The fitted curve (magenta line) has a slope of 2 in the log-log plot, indicating quadratic scaling of the THz SHG with respect to the fundamental radiation field strength.

3 THz SHG polarization dependence

3.1 Second harmonic generation

To demonstrate the origin of SHG due to the USMR effect, we consider the propagation of THz light in a material with conductivity (σ) that varies with the incident THz radiation. Assuming that the modulation of conductivity induced by the THz field is a small contribution, the total conductivity can be expressed as: $\sigma\left(E_{THz}\right) = \sigma_0 + \sigma_1 E_{THz}$, where $\sigma_1 E_{THz}$ represents the component of conductivity that is linearly dependent on the THz field strength, and σ_0 denotes the THz-field-independent contribution. The permittivity of conducting materials can be expressed as: $\epsilon = \Re\epsilon - \frac{i\sigma}{\Omega\epsilon_0}$, where $\Re$ denotes the real part of the permittivity and ϵ_0 is the vacuum permittivity. Considering the THz-field-dependent contribution to the sample conductivity, we can reformulate the sample permittivity as follows:

$$\epsilon\left(E_{THz}\right) = \Re\epsilon - \frac{i\sigma_0}{\Omega\epsilon_0} - \frac{i\sigma_1 E_{THz}}{\Omega\epsilon_0} = \tilde{\epsilon} - \frac{i\sigma_1 E_{THz}}{\Omega\epsilon_0}, \tag{1}$$

here $\tilde{\epsilon}$ is the stationary permittivity of the sample (without the THz field applied). Solving the Maxwell equations gives us the wave propagation equations [1]:

$$\left(\Delta - \frac{\tilde{\epsilon}}{c}\frac{\partial^2}{\partial t^2}\right) E_{THz} = \frac{i\sigma_1 E_{THz}^2}{\epsilon_0^2 c^2 \Omega}. \tag{2}$$

The right side of Eq.(2) corresponds to the light pulse propagation within the medium, while the left side describes the nonlinear effects in the medium. By neglecting the propagation effects (assuming $\tilde{\epsilon} = 1$) and applying the Slowly Varying Envelope Approximation [1], the solution of the Eq.(2) represents the THz SHG with an amplitude $E\left(2\Omega\right) \sim \sigma_1 E_{THz}^2 = \Delta\sigma E_{THz}$. Here $\Delta\sigma = \sigma_1 E_{THz}$ denotes the THz field-induced modulation of the sample's electrical conductivity, which, in our case, arises from the unidirectional spin Hall magnetoresistance. Given that the modulation of the interface resistivity constitutes a small fraction of the sample's overall resistivity, we can express it as: $\left(\rho^{\uparrow\uparrow} - \rho^{\downarrow\uparrow}\right) \sim \Delta\sigma$, leading to: $E\left(2\Omega\right) \sim \left(\rho^{\uparrow\uparrow} - \rho^{\downarrow\uparrow}\right) E_{THz}$.

3.2 Linearly polarised light

Let's consider linearly polarised THz pulses illuminating the heterostructure comprising ferromagnetic and heavy metal layers (as depicted in Fig. 1 in the main text). For simplicity, we assume that the sample's magnetization lies in-plane at an angle ψ with respect to the x-axis. The THz pump polarization is oriented at an angle ϕ in relation to the x-axis. The (x, y) plane coincides with the sample surface. The THz fields are along the x and y axis:

$$\mathbf{E}_{THz} = E_0 e^{i\Omega t}\begin{pmatrix} E_x \\ E_y \end{pmatrix} = E_0 e^{i\Omega t}\begin{pmatrix} \cos\phi \\ \sin\phi \end{pmatrix}$$

The sample temperature is modulated by the THz peak intensity, which is proportional to $\delta T \sim \left(\mathbf{E}_{THz}\right)^2 = E_0^2 + E_0^2 e^{i2\Omega t}$. The second term represents the modulation

of the sample temperature at the frequency of the second harmonic of the excitation pulse frequency. The temperature modulation of the sample induces spin currents propagating along the z-axis with spin polarisation along the sample magnetisation $\mathbf{m} = \begin{pmatrix} \cos\psi \\ \sin\psi \end{pmatrix}$. The spin currents in the Pt layer are converted into electric currents by the inverse spin Hall effect and have the polarisation as

$$E_{ISHE} \sim \alpha_{ISHE}\delta T \left[\mathbf{e}_z, \mathbf{m}\right] = \alpha_{ISHE}\delta T \begin{pmatrix} -\sin\psi \\ \cos\psi \end{pmatrix}.$$

α_{ISHE} - coefficient of the inverse spin Hall effect in the platinum layer. $\mathbf{E}_{ISHE}$ does not depend on the THz excitation polarisation and is orthogonally polarised with respect to the sample magnetisation.

The effect of USMR can be considered by introducing a resistance modulation defined as $\delta R \sim (\mathbf{s}, \mathbf{m})$, where $\mathbf{s}$ is the spin accumulation at the interface due to the spin Hall effect. The spin accumulation can be expressed as follows:

$$\mathbf{s} \sim [\mathbf{E}, \mathbf{e}_z] = e^{i\Omega t} \begin{pmatrix} -\sin\phi \\ \cos\phi \end{pmatrix}.$$

The USMR contribution to the sample resistivity is

$$\delta R = \delta R_0 E^{i\Omega t} \cos\left(\phi - \psi\right),$$

which is maximal when $\mathbf{s}$ is anti- or collinear with $\mathbf{m}$ and is zero for orthogonal orientation. The resistivity is modulated at the Ω frequency. The incident THz radiation at Ω frequency scattering on the sample resistivity modulation at Ω frequency will generate THz second harmonic generation at 2Ω frequency with electric field defined as:

$$\mathbf{E}_{USMR} = \delta R \mathbf{E}_{THz} = E_0^2 e^{2i\Omega t} \begin{pmatrix} \cos\phi\cos\left(\phi - \psi\right) \\ \sin\phi\cos\left(\phi - \psi\right) \end{pmatrix}.$$

The $\mathbf{E}_{USMR}$ is polarised along the THz pump pulse polarisation and its amplitude scales as a projection of $\mathbf{s}$ on $\mathbf{m}$.

3.3 Elliptically polarised light

Let's assume an ideal quarter-wave plate in the path of the THz pump, with the easy axis oriented at an angle χ relative to the x-axis. The THz light passing through the waveplate can be expressed as

$$\mathbf{E}_{THz} = \frac{1}{\sqrt{1 + a^2}} \begin{pmatrix} \cos\Omega t \\ a\sin\Omega t \end{pmatrix},$$

where $a = \sin 2\chi$ corresponds to the beam ellipticity.

The THz instantaneous intensity is $I_{THz} \sim \frac{1}{1+a^2}\left(\cos^2\Omega t + a^2\sin^2\Omega t\right)$. The modulation of the instantaneous intensity at the THz frequency 2Ω, which is responsible for the THz SHG via $\mathbf{E}_{ISHE}$, can be expressed as:

$$\mathbf{E}_{ISHE} \sim E_0^2 \frac{1-a^2}{1+a^2} \begin{pmatrix} -\sin\psi \\ \cos\psi \end{pmatrix} \sin 2\Omega t.$$

$\mathbf{E}_{ISHE}$ is maximal for linear polarised excitation ($a = 0$), and is zero for circular polarised light ($a = 1$).

Similarly to the case of linear polarised excitation, it can be shown that for elliptically polarised light the magnetoresistive contribution can be expressed as follows:

$$\delta R = \frac{1}{\sqrt{1+a^2}} \left(\cos\Omega t \sin\psi - a \sin\Omega t \cos\psi\right).$$

The corresponding

$$\mathbf{E}_{USMR} = \frac{E_0^2}{1+a^2} \left(\cos\Omega t \sin\psi - a \sin\Omega t \cos\psi\right) \begin{pmatrix} \cos\Omega t \\ a \sin\Omega t \end{pmatrix}.$$

4 References

[1] Boyd, R.W., Gaeta, A.L., Giese, E. (2023). Nonlinear Optics. In: Drake, G.W.F. (eds) Springer Handbook of Atomic, Molecular, and Optical Physics. Springer Handbooks. Springer, Cham.
